# More than a decade of real-world experience of pegvisomant for acromegaly: ACROSTUDY

**DOI:** 10.1530/EJE-21-0239

**Published:** 2021-08-03

**Authors:** Maria Fleseriu, Dagmar Führer-Sakel, Aart J van der Lely, Laura De Marinis, Thierry Brue, Joli van der Lans-Bussemaker, Judith Hey-Hadavi, Cecilia Camacho-Hubner, Michael P Wajnrajch, Srinivas Rao Valluri, Andrew Anthony Palladino, Roy Gomez, Roberto Salvatori

**Affiliations:** 1 Pituitary CenterDivision of Endocrinology, Diabetes, & Clinical Nutrition, Department of Medicine; 2 Pituitary Center, Department of Neurological Surgery, Oregon Health & Science University, Portland, Oregon, USA; 3 Department of Endocrinology Diabetology and Metabolism, Endocrine Tumour Center at West German Cancer Center, University Hospital Essen, University of Duisburg-Essen, Essen, Germany; 4 Division of Endocrinology, Department of Internal Medicine, Erasmus Medical Centre, Rotterdam, Netherlands; 5 Pituitary Unit, Department of Endocrinology, Fondazione A Gemelli, IRCCS, Università Cattolica del Sacro Cuore, Rome, Italy; 6 Assistance Publique-Hôpitaux de Marseille, Hopital de la Conception, and Aix-Marseille Université, Marseille Medical Genetics, Marseille, France; 7 Pfizer, Capelle aan den IJssel, Netherlands; 8 Pfizer, New York, New York, USA; 9 Division of Pediatric Endocrinology, Department of Pediatrics, New York University Langone Medical Center, New York, New York, USA; 10 Pfizer, Collegeville, Pennsylvania, USA; 11 Pfizer, Brussels, Belgium; 12 Division of Endocrinology and Pituitary Center, Johns Hopkins University School of Medicine, Baltimore, Maryland, USA

## Abstract

**Objective:**

To report the final long-term safety and efficacy analyses of patients with acromegaly treated with pegvisomant from the ACROSTUDY.

**Design:**

Global (15 countries), multicentre, non-interventional study (2004–2017).

**Methods:**

The complete ACROSTUDY cohort comprised patients with acromegaly, who were being treated with pegvisomant (PEGV) prior to the study or at enrolment. The main endpoints were long-term safety (comorbidities, adverse events (AEs), pituitary tumour volumes, liver tests) and efficacy (IGF1 changes).

**Results:**

Patients (*n* = 2221) were treated with PEGV for a median of 9.3 years (range, 0–20.8 years) and followed up for a median of 7.4 years (range, 0–13.9 years). Before PEGV, 96.3% had received other acromegaly treatments (surgery/radiotherapy/medications). Before PEGV treatment, 87.2% of patients reported comorbidities. During ACROSTUDY, 5567 AEs were reported in 56.5% of patients and of these 613 were considered treatment-related (in 16.5% of patients) and led to drug withdrawal in 1.3%. Pituitary imaging showed a tumour size increase in 7.1% of patients; the majority (71.1%) reported no changes. Abnormal AST or ALT liver tests occurred in 3.2% of patients. IGF1 normalization rate improved over time, increasing from 11.4% at PEGV start to 53.7% at year 1, and reaching 75.4% at year 10 with the use of ≥30 mg PEGV/day in an increasing proportion of patients.

**Conclusion:**

This comprehensive review of the complete cohort in ACROSTUDY confirmed the overall favourable benefit-to-risk profile and high efficacy of PEGV as mono- and combination therapy in patients with an aggressive course/uncontrolled/active acromegaly requiring long-term medical therapy for control.

## Introduction

Acromegaly is a rare endocrine disorder that develops when the pituitary gland produces excess growth hormone (GH) during adulthood and is mostly caused by a GH-secreting pituitary adenoma (1). Circulating GH triggers the overproduction of insulin-like growth factor-1 (IGF1), which in turn stimulates the growth of cartilage, soft tissues, and organs causing somatic overgrowth and disfigurements (1, 2). Some consequences, such as insulin resistance, are due to the direct effect of GH (3). If not treated adequately, acromegaly can lead to serious comorbidities and may become life-threatening. The increased mortality rate in acromegaly is often due to co-existing cardiovascular, metabolic, and respiratory diseases (2, 4).

Multidisciplinary treatment approaches for acromegaly include surgery, radiotherapy, and medications (5, 6). Transsphenoidal surgery may offer rapid GH reduction and is recommended as the primary therapy when surgical cure is possible or for tumour debulking (1). Radiotherapy, with a slow response onset and high risk of hypopituitarism (4), is reserved for patients with postoperative residual tumour mass when medical therapy fails to control (1, 7). Medications include somatostatin receptor ligands (SRLs), dopamine agonists (DAs), and the GH receptor antagonist, pegvisomant (PEGV). Long-acting octreotide and lanreotide are commonly used SRLs to suppress GH secretion, IGF1, and reduce tumour size in acromegaly patients (8, 9). Pasireotide is a newer SRL, which can confer additional biochemical control in some patients nonresponsive to octreotide or lanreotide (10). DAs, such as cabergoline, have limited efficacy and are often used as adjuvant medical therapy (1, 11).

PEGV is a recombinant protein that structurally resembles WT human GH and its recombinant variants (12). It binds to the human GH receptor with greater affinity than native GH and blocks signal transduction, thus reducing circulating IGF1 concentrations. Pegylation of the drug increases its biological half-life. PEGV is administered by s.c. injections and is mainly used as a monotherapy but can also be used in combination with SRLs or DAs. The main biochemical marker to monitor the efficacy of PEGV is serum IGF1 (4, 13).

In the initial pivotal trials, PEGV was generally well tolerated in patients with acromegaly treated for up to 18 months, and serum IGF1 normalization was achieved in 97% of patients with ≥12 months of daily PEGV (14, 15). PEGV was approved in Europe in 2002 (16) and in the United States in 2003 (17) for patients with acromegaly that could not be adequately controlled by surgery and/or radiation therapy and/or medication. The drug was later approved in the United States for use as a primary medical therapy when surgery and/or radiation therapy fail to control the disease or when these therapies are not appropriate (17).

The global, multicentre, non-interventional ACROSTUDY was established in 2004 as post-authorization safety surveillance (PASS) requested by the European Medicines Agency (EMA) to evaluate the long-term safety and efficacy outcomes of PEGV as prescribed in routine clinical practice for acromegaly. Previous reports revealed reassuring safety outcomes in monotherapy and combination therapy (18, 19, 20, 21, 22, 23, 24, 25, 26). With ACROSTUDY concluded in December 2017, this report presents the final safety and efficacy outcomes in the complete cohort of 2221 patients with up to 14 years of follow-up.

## Methods

### Study design

Patients were enrolled in ACROSTUDY on an ongoing basis and followed for a minimum of 5 years. The PASS commitment of evaluating the safety of 5-year treatment with PEGV in at least 1000 patients with acromegaly was fulfilled in 2013 (19, 20). The study was extended as a voluntary PASS (voluntary extension) to study safety (especially changes in glucose) and quality of life measures, which allowed approximately 400 patients already enrolled in the ACROSTUDY to continue (rollover patients), and was open to an additional 100 new patients with acromegaly who were either treatment naïve or semi-naïve (no PEGV treatment within 6 months prior to enrolment) at study entry. The study took place in 15 countries where PEGV was authorized for acromegaly treatment. Treatment doses and schedules were at the discretion of the treating investigators. Patients were assessed during routine clinical practice and followed up until December 2017, when ACROSTUDY was terminated.

The ACROSTUDY data reported here were collected in compliance with, and consistent with, the most recent version of the Declaration of Helsinki. In addition, the study adhered to all applicable local laws and regulatory requirements in the countries involved. Local ethical approval was obtained for all participating centers (see Supplementary Appendix for list of ethics committees and institutional review boards, see section on supplementary materials given at the end of this article), and all patients provided written informed consent before any data were captured.

### Patients

This report focused on the full analysis population, which included all patients who were enrolled in ACROSTUDY from 2004 to December 2017 and received at least one dose of PEGV. Patients with acromegaly who were being treated or just starting PEGV treatment prior to enrolling in ACROSTUDY were included. Some European study sites were able to enrol paediatric patients (<18 years). Pituitary imaging within 6 months prior to study enrolment was recommended for all patients. During the voluntary extension, only adult patients (≥18 years) were eligible for enrolment. Patients were excluded from enrolment if they had participated in any other investigational trial for acromegaly in the previous 6 months, required surgery to decompress the tumour or non-medical therapy due to visual field loss, cranial nerve palsies or intracranial hypertension, or had allergies to PEGV or its ingredients. Women who were pregnant or lactating were also not enrolled. In the voluntary PASS, re-enrolment of patients who were discontinued from the ACROSTUDY was not allowed. Informed consent was obtained from all patients.

### Safety

The baseline was defined as the start of PEGV treatment. Safety data included adverse events (AEs), liver tests and pituitary tumour imaging. All reported AEs, serious AEs (SAEs), AEs of special interest (e.g. administration-site reactions, hepatobiliary-related AEs, and changes in tumour size), and deaths were assessed. All AEs were coded using the Medical Dictionary for Regulatory Activities dictionary (MedDRA version 20.1). Concomitant medication and acromegaly-related comorbidities were collected from treating physicians using a standardized questionnaire. Any disorders with onset after enrolment were reported as AEs. Safety endpoints related to liver tests included the percentage of patients with elevated transaminases (≥3-fold of upper limit of normal (ULN)) in the alanine aminotransferase (ALT) or aspartate aminotransferase (AST) tests by visit window and the percentage of patients with any liver test elevation (alkaline phosphatase, bilirubin, gamma-glutamyltransferase; ≥ULN). Pituitary tumour images were collected and read locally at baseline, at months 6 and 12 post-treatment, and annually as determined by the investigator. A significant change in tumour size was defined as a >3 mm change in the largest diameter of a pituitary microadenoma or a >20% change in tumour volume of a macroadenoma (>10 mm). If significant changes were determined by local radiologists, all available images for that patient were requested to be re-assessed by a central reader (this was not required during the voluntary extension). When available, assessments of pituitary tumour volume (increased, decreased, or unchanged) relative to baseline were summarized by visit window.

### Efficacy

Serum IGF1 levels were measured at local and central laboratories. Efficacy data analysed included the proportion of patients by IGF1 values (<lower limit of normal (LLN), normal, >ULN according to the laboratory reference values) and proportion of patients who achieved and maintained IGF1 level within the normal range. An 'IGF1 controlled' status was assigned to those with a value in the normal range from their latest IGF1 measurement. An IGF1- status of 'not controlled' was assigned to those with an IGF1 value higher than ULN or lower than LLN. Fasting blood glucose and HbA1c parameters were obtained through routine clinical practice and percentages of patients with values outside of the normal range were summarized.

### Statistical analysis

No pre-specified statistical hypotheses were tested in the study. All data were summarized with descriptive statistics. Percentages of patients experiencing an AE, liver test elevation, change in tumour size, or other laboratory value outside of the normal range were assessed for a specified time period or time point. IGF1 levels were categorized according to the laboratory reference values (normal, >ULN, or <LLN) and summarized by years of PEGV treatment with mean daily dose included. Comorbidities were quantified as reported by the treating physicians.

## Results

### Demographics

A total of 2221 patients from 14 European countries and the United States (Table 1) participated in ACROSTUDY and were included in the safety population. Of these, 434 were rollover patients and 110 were naïve/semi-naïve patients, which were enrolled as part as the voluntary PASS extension. The majority of patients were Caucasian (92.4%), and the percentage of male to female was similar. Overall, patients had a median age of 41.1 years at acromegaly diagnosis, 49.7 years at the start of PEGV treatment, and 51.5 years at study enrolment. The maximum duration of PEGV treatment was 20.8 years, with a median of 9.3 years. Patients were followed in ACROSTUDY for up to 13.9 years (median: 7.4 years). A total of 11 paediatric patients were enrolled (0.5%); 5 were between 2 and 11 years old and 6 were 12 to <18 years old. Pituitary function data at baseline showed deficiencies for follicle-stimulating hormone/luteinizing hormone (FSH/LH) in 37.8% of patients, thyroid-stimulating hormone (TSH) in 28.9%, and adrenocorticotropic hormone (ACTH) in 28.2% (Table 2).

**Table 1:** Patient demographic and characteristics. Data were reported as median (min, max) unless indicated otherwise.

	Male (*n* = 1129)	Female (*n* = 1092)	Total (*n* = 2221)
Age at, years			
Diagnosis	39.6 (1.7−83.7)^a^	42.8 (2.6−81.0)^b^	41.1 (1.7−83.7)^c^
Start	48.1 (15.2−85 .6)	51.7 (3.9−85.0)	49.7 (3.9−85.6)
ACROSTUDY start	49.7 (15.2−86.5)	53.0 (3.9−89.8)	51.5 (3.9−89.8)
Years in ACROSTUDY, years	7.4 (0.0−13.7)	7.4 (0.0−13.9)	7.4 (0.0−13.9)
Duration on PEGV, years	9.3 (0.0−20.8)	9.3 (0.0−19.3)	9.3 (0.0−20.8)
Prior treatment for acromegaly, *n* (%)			
Treatment received			2138 (96.3)
Medical therapy only			418 (18.8)
Radiation only			1 (0.0)
Surgery only			91 (4.1)
Medical and radiation			44 (2.0)
Medical and surgery			1069 (48.1)
Radiation and surgery			36 (1.6)
Medical, surgery and radiation			479 (21.6)
Unknown			53 (2.4)
Country, *n* (%)			
Germany	284 (25.2)	264 (24.2)	548 (24.7)
Italy	232 (20.5)	234 (21.4)	466 (21.0)
France	165 (14.6)	147 (13.5)	312 (14.0)
United States	111 (9.8)	96 (8.8)	207 (9.3)
Spain	86 (7.6)	114 (10.4)	200 (9.0)
Netherlands	97 (8.6)	78 (7.1)	175 (7.9)
Greece	14 (1.2)	42 (3.8)	56 (2.5)
Sweden	29 (2.6)	20 (1.8)	49 (2.2)
Great Britain	28 (2.5)	20 (1.8)	48 (2.2)
Slovakia	18 (1.6)	24 (2.2)	42 (1.9)
Denmark	21 (1.9)	17 (1.6)	38 (1.7)
Belgium	18 (1.6)	19 (1.7)	37 (1.7)
Austria	13 (1.2)	10 (0.9)	23 (1.0)
Portugal	8 (0.7)	5 (0.5)	13 (0.6)
Hungary	5 (0.4)	2 (0.2)	7 (0.3)
Race			
Caucasian	1042 (92.3)	1011 (92.6)	2053 (92.4)
Black	7 (0.6)	9 (0.8)	16 (0.7)
Asian^d^	13 (1.2)	15 (1.4)	28 (1.3)
Hispanic	6 (0.5)	5 (0.5)	11 (0.5)
African American	2 (0.2)	0	2 (0.1)
Other	29 (2.6)	19 (1.7)	48 (2.2)
Missing	30 (2.7)	33 (3.0)	63 (2.8)

^a^*n* = 1126, ^b^*n* = 1081, ^c^*n* = 2207, ^d^included race as 'Oriental'.

**Table 2:** Pituitary function test at the start of pegvisomant treatment.

	Total (*n*)	Deficiency *n* (%)	Normal for age *n* (%)	Hypersecretion *n* (%)
FSH/LH (Gonadal hormone)	619	234 (37.8)	375 (60.6)	10 (1.6)
ACTH	560	158 (28.2)	399 (71.3)	3 (0.5)
TSH	646	187 (28.9)	455 (70.4)	4 (0.6)
ADH (Diabetes insipidus)	409	9 (2.2)	395 (96.6)	5 (1.2)
Prolactin	592	31 (5.2)	489 (82.6)	72 (12.2)
Other hormones	49	4 (8.2)	43 (87.8)	2 (4.1)
Without HCG	7	3 (42.9)	3 (42.9)	1 (14.3)

ACTH, adrenocorticotropic hormone; ADH, antidiuretic hormone; FSH, follicle-stimulating hormone; HCG, human chorionic gonadotropin; LH, luteinizing hormone; TSH, thyroid-stimulating hormone.

Before PEGV initiation, most patients (87.2%) presented at least one comorbidity (Table 3). The most commonly reported comorbidities were hypertension (51.3%), diabetes mellitus (32.2%), osteoarthritis (21.3%), and sleep apnoea (20.8%). Between PEGV start and ACROSTUDY entry, 30.1% of 1586 patients reported new comorbidities, with hypertension (12.3%), osteoarthritis (10.7%), and diabetes mellitus (10.5%) being the most common. Neoplasia (benign or malignant) was reported in 38.8% of patients at baseline (Table 3), most commonly in the thyroid (17.6%) and colon (14.8%).

**Table 3:** Comorbidities (first occurrence) reported during different time periods of ACROSTUDY.

Number (%) of patients	Before PEGV start (*n* = 2221)	Between PEGV start and ACROSTUDY entry (*n* = 1586)	After ACROSTUDY start^a^ (*n* = 2221)
Patients with ≥1 comorbidities^b^	1937 (87.2)	478 (30.1)	172 (7.7)
Patients with no comorbidities	102 (4.6)	179 (11.3)	N/A
Patients with missing report	182 (8.2)	929 (58.6)	N/A
Metabolic	624 (32.2)	50 (10.5)	41 (23.8)
Diabetes mellitus	624 (32.2)	50 (10.5)	41 (23.8)
Cardiovascular	1068 (55.1)	108 (22.6)	26 (15.1)
Hypertension	993 (51.3)	59 (12.3)	18 (10.5)
Arrhythmia	116 (6.0)	26 (5.4)	7 (4.1)
Cardiomyopathy	148 (7.6)	22 (4.6)	1 (0.6)
Myocardial infarction	38 (2.0)	6 (1.3)	1 (0.6)
Coronary heart disease	79 (4.1)	5 (1.0)	0
Coronary artery bypass surgery	18 (0.9)	2 (0.4)	0
Coronary angioplasty with or without stent	33 (1.7)	3 (0.6)	0
Cerebrovascular	42 (2.2)	8 (1.7)	2 (1.2)
Transient ischemic attack	21 (1.1)	5 (1.0)	1 (0.6)
Infarction	17 (0.9)	2 (0.4)	1 (0.6)
Haemorrhage	5 (0.3)	1 (0.2)	0
Respiratory	497 (25.7)	47 (9.8)	13 (7.6)
Sleep apnoea	403 (20.8)	29 (6.1)	4 (2.3)
COPD	66 (3.4)	4 (0.8)	2 (1.2)
Other respiratory disease	109 (5.6)	15 (3.1)	7 (4.1)
Musculoskeletal	642 (33.1)	76 (15.9)	19 (11.0)
Osteoarthritis	412 (21.3)	51 (10.7)	13 (7.6)
Osteoporosis	75 (3.9)	15 (3.1)	3 (1.7)
Surgery for carpal tunnel syndrome	201 (10.4)	4 (0.8)	1 (0.6)
Other surgery for musculoskeletal disease	110 (5.7)	9 (1.9)	2 (1.2)
Liver/gallbladder	429 (22.1)	87 (18.2)	9 (5.2)
Hepatic disease	137 (7.1)	59 (12.3)	5 (2.9)
Surgery for gallstones	331 (17.1)	34 (7.1)	4 (2.3)
Tumours	751 (38.8)	136 (28.5)	33 (19.2)
Thyroid	341 (17.6)	35 (7.3)	10 (5.8)
Benign	286 (14.8)	29 (6.1)	9 (5.2)
Malignant	40 (2.1)	6 (1.3)	1 (0.6)
Unknown/missing	15 (0.8)	0	0
Colon	286 (14.8)	46 (9.6)	12 (7.0)
Adenomatous polyps	192 (9.9)	32 (6.7)	10 (5.8)
Other benign tumour	61 (3.1)	11 (2.3)	2 (1.2)
Adenocarcinoma	13 (0.7)	2 (0.4)	0
Other malignant tumour	5 (0.3)	1 (0.2)	0
Missing	15 (0.8)	0	0
Breast	59 (3.0)	8 (1.7)	1 (0.6)
Benign	33 (1.7)	4 (0.8)	1 (0.6)
Malignant	24 (1.2)	3 (0.6)	0
Unknown/missing	2 (0.1)	1 (0.2)	0
Prostate	45 (2.3)	12 (2.5)	2 (1.2)
Benign	36 (1.9)	6 (1.3)	1 (0.6)
Malignant	5 (0.3)	5 (1.0)	1 (0.6)
Unknown/missing	4 (0.2)	1 (0.2)	0
Skin	36 (1.9)	12 (2.5)	3 (1.7)
Benign	24 (1.2)	7 (1.5)	1 (0.6)
Malignant	10 (0.5)	3 (0.6)	2 (1.2)
Unknown/missing	2 (0.1)	2 (0.4)	0
Lung	11 (0.6)	4 (0.8)	2 (1.2)
Benign	5 (0.3)	1 (0.2)	1 (0.6)
Malignant	4 (0.2)	1 (0.2)	1 (0.6)
Unknown/missing	2 (0.1)	2 (0.4)	0
Other comorbidity	1159 (59.8)	138 (28.9)	45 (26.2)
Other clinically significant comorbidity	1012 (52.2)	125 (26.2)	45 (26.2)
Goitre	100 (5.2)	2 (0.4)	0
Headache	47 (2.4)	11 (2.3)	0

^a^Per protocol, comorbidities onset after ACROSTUDY start were reported as adverse events. ^b^Patients could have had >1 comorbidity. Percentages for comorbidities calculated using this number as denominator.

COPD, chronic obstructive pulmonary disease; PEGV, pegvisomant.

### Acromegaly treatment

**Figure 1:**
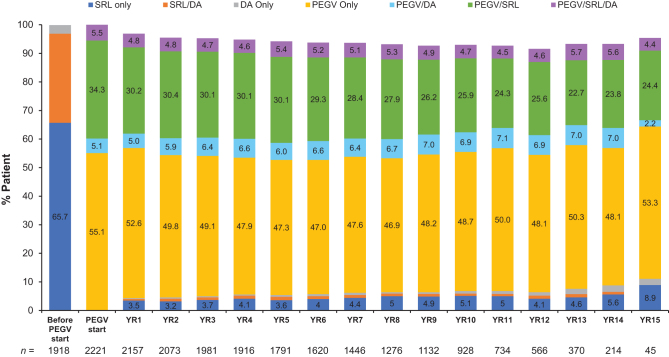
Medications received for acromegaly prior to and during the years of PEGV treatment. *n* indicates the number of patients with available data. DA, dopamine agonist; PEGV, pegvisomant; SRL, somatostatin receptor ligand.

Prior to PEGV treatment, almost half (48.1%) of patients had been treated with both medical and surgical therapies, 21.6% had all three interventions (surgery, radiotherapy, and medication) and 18.8% medication alone (Table 1). Before PEGV start, 65.7% of patients received SRLs only, 31.2% received SRLs combined with DAs and 3.0% used DAs alone (Fig. 1). At PEGV start, the most commonly prescribed treatment was PEGV monotherapy (55.1%), followed by PEGV combined with an SRL (34.3%; Fig. 1). Treatment pattern slightly changed over time, with the use of PEGV monotherapy ranging between 47.0 and 55.1%, while the use of PEGV/SRL decreased from 34.3 to 24.4% over the years.

**Figure 2:**
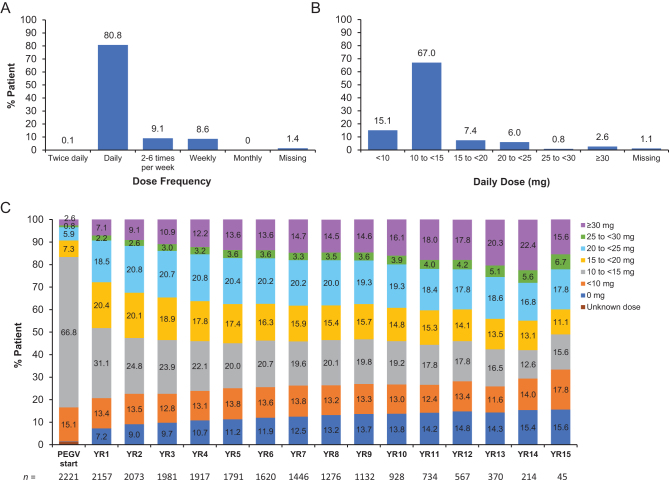
Dose frequency (A) and daily dose (B) of pegvisomant (PEGV) at initiation and (C) administered dose of PEGV by year (daily dose).

Most patients initiated PEGV treatment with a daily dose (80.8%) with the 10 to <15 mg dose being the most common (67.0%; Fig. 2). Some patients received <10 mg PEGV doses, two to six times per week (7.7%) or weekly (7.1%). The most common PEGV doses at year 1 were 10 to <15 mg (31.1%), 15 to <20 mg (20.4%) and 20 to <25 mg (18.5%). Overall, PEGV doses were titrated up over time. Users of ≥30 mg PEGV daily increased from 7.1% at year 1 to 22.4% at year 14.

### Safety

#### Adverse events

Among the 2221 patients enrolled in ACROSTUDY, 5567 AEs were reported in 1255 patients (56.5%; Table 4), with the most commonly reported AEs being increased IGF1 (10.4%), headache (5.1%), vitamin D deficiency (4.9%), arthralgia (4.6%), osteoarthritis (3.6%), depression (2.6%), diabetes mellitus (2.3%), cholelithiasis (2.3%), and colonic polyp (2.2%). Only 613 of the AEs were considered treatment-related and were reported for 16.5% of patients (Table 4). The most common treatment-related AEs (≥1%) were increased IGF1 levels (1.9%), increased transaminases levels (1.5%), lipohypertrophy (1.2%), and decreased IGF1 levels (1.1%).

**Table 4:** Adverse events and deaths in full analysispPopulation (*n* = 2221).

	All-causality *n* (%)	Treatment-related *n* (%)
Number of AEs	5,567	613
Patients with AEs	1255 (56.5)	367 (16.5)
Drug withdrawn^a^ due to AEs	256 (11.5)	28 (1.3)
Patients with SAEs	523 (23.5)	53 (2.4)
Drug withdrawn^a^ due to SAEs	167 (7.5)	28 (1.3)
Dose reduced due to SAEs	7 (0.3)	2 (0.1)
Death	87 (3.9)	0
Patients with AEs of special interest		
Administration-site condition AEs	78 (3.5)	71 (3.2)
Hepatobiliary-related AEs	225 (10.1)	98 (4.4)
Pituitary tumour AEs^b^	96 (4.3)	24 (1.1)

^a^Withdrawal could be temporary, permanent or delayed. ^b^Change in tumour size AEs.

AE, adverse event; SAE, serious adverse event.

SAEs were reported for 523 patients (23.5%) and considered treatment-related in 53 patients (2.4%). The most frequently reported treatment-related SAEs were recurrent (0.4%; *n* = 8) or benign (0.3%; *n* = 6) pituitary tumour, any elevated liver test values (0.6%; *n* = 14) and hepatobiliary disorders (0.3%; *n* = 6). Drug withdrawal (temporary, permanent, or delayed) due to AEs occurred in 256 (11.5%) patients, and due to SAEs in 167 (7.5%) patients. Of all discontinuations, only a small proportion were due to treatment-related AEs (1.3%) or SAEs (1.3%).

AEs of special interest related to the administration-site condition were reported in 3.5% of patients, with most of them related to PEGV treatment (3.2%; Table 4). Most common treatment-related AEs were lipohypertrophy (reported under skin and s.c. tissue disorders), for 1.2% (*n* = 27) of patients and injection-site reaction AEs, for 0.8% (*n* = 18) of patients. Administration-site condition AEs led to PEGV withdrawal (or dose reduction) in 1.1% of patients, with lipodystrophy/lipohypertrophy being the cause 13 times.

Overall, 87 deaths were reported, none of which were reported as treatment-related (Table 4). Common causes for death included cardiac failure (*n* = 6), cerebrovascular event (*n* = 3), myocardial infarction (*n* = 3), respiratory failure (*n* = 3), and cardiac arrest/sudden cardiac death (*n* = 4).

#### Pituitary tumour imaging

**Figure 3:**
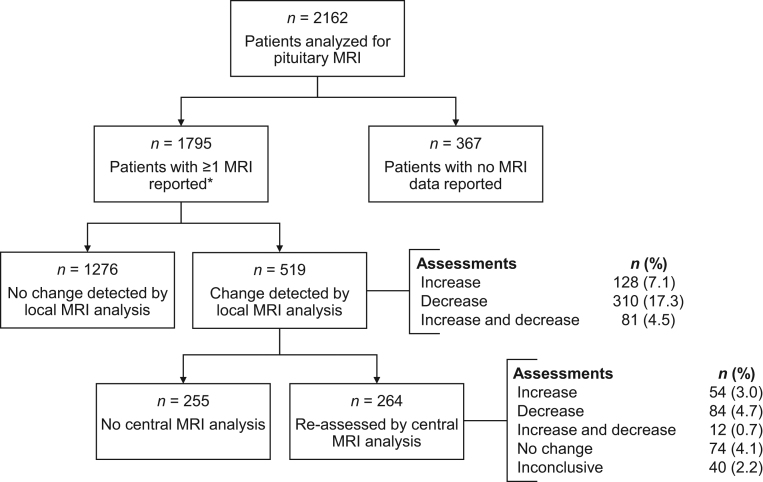
Number of patients with local and central MRI analysis. *At least one pituitary imaging result was reported ≥30 days after treatment initiation.

The percentage of patients receiving a pituitary MRI scan was 40.3% at year 1 and 45.2% at year 2, which decreased over time to 31.0% at year 5 and 4.4% at year 15. A total of 1795 patients had ≥1 pituitary imaging result after PEGV initiation (Fig. 3) and of these, 1276 (71.1%) had no change in pituitary tumour size detected by local MRI analysis. Changes in pituitary tumour size relative to baseline or the last examination were detected at least once by local assessment in 519 patients, including 128 (7.1%) with an increase only, 310 (17.3%) with a decrease only, and 81 (4.5%) with both an increase and decrease (observed at different timepoints). As per the protocol, investigators were asked to send MRIs for central analysis if significant changes were determined by local radiologists (not required during the voluntary extension). For the 264 out of 519 patients, MRI results were re-assessed by central reading, which showed tumour volume increases in 54 (3.0%) patients, decreases in 84 (4.7%) patients, both increases and decreases in 12 (0.7%) patients, no change in 74 (4.1%) patients, and insufficient data in 40 (2.2%) patients. Changes in pituitary tumour size were reported as an AE of special interest in 4.3% of patients (Table 4) and caused 1.4% of patients to discontinue PEGV treatment.

#### Liver tests

A total of 3.2% of 2221 patients had an ALT/AST value of >3× ULN at any time point during PEGV treatment (Table 5). Of the 1327 patients with normal baseline ALT/AST values, 42 (3.2%) had values that increased to >3× ULN during treatment. Increased ALT/AST/transaminases led to the withdrawal of PEGV treatment (or dose reduction) occurred 19 times. Overall, for 10.1% of patients, hepatobiliary-related AEs were reported (Table 4), which led to PEGV withdrawal in 1.7% of patients.

**Table 5:** Shift table of liver tests (ALT or AST) measured at baseline or at any time point during the course of pegvisomant (PEGV) treatment.

Baseline	*N*^e^	During PEGV treatment			
		Normal^b^ *n* (%)	1× to 3× ULN^c^ *n* (%)	>3× ULN^d^ *n* (%)	Missing^a^ *n* (%)
Normal	1327	828 (62.4)	333 (25.1)	42 (3.2)	124 (9.3)
1× to 3× ULN	115	37 (32.2)	60 (52.2)	9 (7.8)	9 (7.8)
>3× ULN	10	4 (40)	4 (40)	2 (20)	0 (0.0)
Missing	769	494 (64.2)	163 (21.2)	18 (2.3)	94 (12.2)
Total	2221	1363 (61.4)	560 (25.2)	71 (3.2)	227 (10.2)

^a^Patients had missing AST and ALT. ^b^Patients had normal AST and normal ALT. ^c^Patients had AST or ALT in the range of 1× ULN to 3× ULN. ^d^Patients had AST or ALT in the range of >3× ULN. ^e^ *N* was used a denominator to calculated percentages in each row.

ALT, alanine aminotransferase test; AST, aspartate aminotransferase test; ULN, upper limit of normal.

No liver failure was reported in the study. Two patients had liver enzyme abnormalities that satisfied potential Hy’s Law criteria (ALT/AST >3× ULN and peak total bilirubin result >2× ULN). However, both had other conditions that were believed by the investigators to have led to their elevated liver test results.

### Efficacy

#### IGF1 normalization

**Figure 4:**
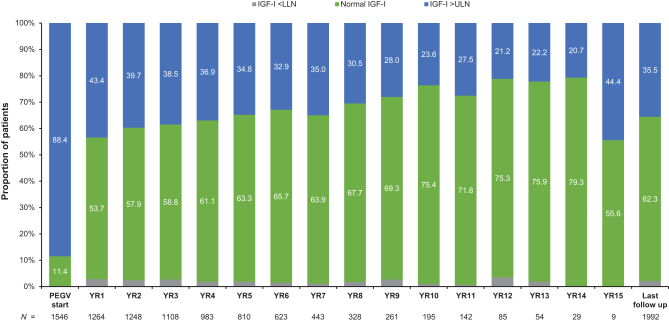
Proportion of patients achieved IGF1 values within or outside the normal range at PEGV start and during the years of PEGV treatment. *n* indicates the number of patients with available data. Last follow up was the last observation after baseline. IGF1, insulin-like growth factor-1; LLN, lower limit of normal; PEGV, pegvisomant; ULN, upper limit of normal.

**Figure 5:**
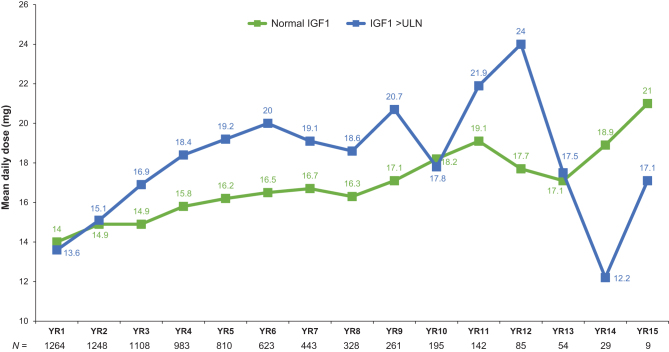
Mean daily dose of PEGV received by patients who achieved IGF1 normalization or who had IGF1 > ULN during the years of PEGV treatment. Last PEGV dose prescribed before the IGF1 examination date was used. *N* indicates the number of patients with available data. Last follow up was the last observation after baseline. IGF1, insulin-like growth factor-1; LLN, lower limit of normal; PEGV, pegvisomant; ULN, upper limit of normal.

At PEGV start, 11.4% of the patients (*n* = 1546) had an IGF1 concentration within the normal reference range, while 88.4% had an IGF-1 > ULN. The percentage of patients with IGF1 levels within the normal range increased from 53.7% at year 1 to 63.3% at year 5, and remained above 60% (63.3–79.3%) throughout most of the study (Fig. 4). Overall, 62.3% of patients with IGF1 data available beyond baseline assessment achieved an IGF1 normalization at the last observation. The degree of IGF1 normalization was accompanied by an increase in PEGV doses; thus, mean daily doses in patients with IGF1 normalization increased from 14.0 mg at year 1 to 18.2 mg at year 10 (Fig. 5). PEGV doses in patients with active acromegaly also increased over time and were higher than those in controlled patients at most years.

#### Diabetes and glucose metabolism

Before the start of PEGV treatment, 624 patients (32.2%) reported having diabetes mellitus. Out of the 996 patients (44.8%) with a glucose value < 200 mg/dL at PEGV start, 29 (2.9%) had a glucose value > 200 mg/dL reported during treatment. Of the 540 patients who had a baseline HbA1c < 6.5%, 80 (14.8%) had at least one HbA1c value > 6.5% reported while receiving PEGV.

## Discussion

The present final report summarizes data on the complete cohort of 2221 patients who participated in ACROSTUDY with a maximum follow-up time of 13.9 years. Overall, PEGV appeared to be well tolerated over a median duration of 9.3 years of use. The main finding of this study was that PEGV had a favourable safety profile in clinical practice, especially concerning pituitary tumour volumes and liver tests. However, it should be noted that most subjects were Caucasian, and the sample size of non-Caucasian subjects was too small to determine a risk-to-benefit profile. We also note that the pharmacokinetic and pharmacodynamic properties of PEGV are not significantly different between Asian and Western patients so we would expect similar results (27, 28, 29, 30).

The prescribing information of PEGV (17) and Endocrine Society guidelines recommend monitoring tumour size and liver tests during the course of PEGV treatment (1) and as such, pituitary tumour growth and elevated liver enzymes were the main safety concerns with PEGV use. In the full cohort, central MRI reading showed tumour size increase in 3.7% (alone or in combination with a decrease), similar to those reported in the German Pegvisomant Observational Study (3.1%) (31) and other reports of ACROSTUDY (2.2–3.2%) (19, 22, 25). This low incidence suggests that PEGV does not promote tumour volume increases. The observed events could indeed represent the result of tumour recurrence/regrowth due to SRL withdrawal or could simply reflect the natural history of an aggressive tumour (32, 33). Interestingly, the incidence of pituitary tumour volume increase based on local MRI readings was higher than that based on central reading (7.2% vs 3.0%), showing the possible difference in assessment by local analysis as noted previously (19). In smaller, real-life studies of PEGV, a higher incidence of tumour growth was reported (6.5–9.4%; central reading not mentioned), close to that assessed by local reading in ACROSTUDY (28, 29, 30, 34). Over time, the percentage of patients with pituitary MRI scans decreased, likely reflecting perceived patient status and standard of care.

We found here a low incidence of liver enzyme elevation during PEGV treatment, not different from previous reports (18, 19, 20, 22, 26). Only 3.2% of patients with normal baseline ALT/AST value had an elevated ALT/AST (>3× of ULN) at any time point during the follow-up. Overall, increases in liver enzymes were transient in most patients and liver failure was not reported. Elevated liver tests in 5.2–9.3% of patients were observed in other real-world studies of PEGV (29, 30, 31). It is noteworthy that 30–40% of patients in ACROSTUDY received combined PEGV/SRLs (with or without other drugs), which could be expected to result in higher rates of transaminase elevation compared with PEGV alone (23, 35, 36). However, possibilities of transient elevations occurring between PEGV initiation and ACROSTUDY start or between scheduled liver tests can not be excluded (23). In addition, the majority of the hepatobiliary-related AEs were considered to have causes other than PEGV and only 1.7% of patients discontinued PEGV due to these events.

PEGV therapy may cause lipodystrophy, a disorder of adipose tissue, at the injection site (37, 38). In ACROSTUDY, most of the administration-site condition AEs (reported for 2.0% of patients) were considered related to PEGV. As lipodystrophy may be associated with escape (loss of biochemical control in patients previously controlled) from PEGV (38), the PEGV injection site should be monitored for lipodystrophy and frequent injection-site changes (25).

We observed that the IGF1 normalization rate progressively improved with PEGV treatment over time: more than half (53.7%) of patients within 1 year and by a maximum of 79.3% at later years. Almost two-thirds of patients with IGF1 data after baseline (62.3%) had documented IGF1 normalization at last follow-up, consistent with previous findings of 63–73% in the two interim ACROSTUDY reports published in 2012 (19) and 2018 (20). While the IGF1 normalization rate was also lower here than in the pivotal clinical trials leading to PEGV approval (14, 15), our findings agree with other real-life studies including the German Pegvisomant Observational, retrospective Brazilian, retrospective Argentinian, and Japanese post-marketing surveillance studies (28, 29, 30, 31). Any discrepancy could be attributed to lack of uniform dose titration, inadequate patient compliance, and different IGF1 assays or lack of titration/normalization criteria used in these real-world settings vs clinical trials, which have more strict trial criteria (18, 20, 31). For example, a higher rate of IGF1 normalization (85%-90%) was observed in two studies of PEGV-treated patients at tertiary care hospitals, where tight dose titrations and close follow-up were more likely (34, 39). In addition, patient selection may be skewed to those who had more aggressive acromegaly as most patients in ACROSTUDY were enrolled at European sites, where PEGV is indicated as a second-line medication; here almost half of patients had failed surgery and other medications and another 20% also underwent radiation (19, 22). Finally, with the improved biochemical control over time, use of PEGV doses higher than 30 mg daily also increased; however, mean doses were similar between patients with controlled and uncontrolled acromegaly.

The ACROSTUDY results also agree with a comprehensive meta-analysis of 45 observational PEGV studies showing an overall rate of disease control of 60.9% (95% CI: 51.8–69.3%) of patients, which increased to 71.7% (95% CI: 64.0–78.4%) in patients using PEGV alone (40). Similarly, the incidence of increased transaminases was estimated at 3.0% (95% CI: 1.7–5.2%) and tumour growth was 7.3% (95% CI: 4.7–11.1%) (40).

In this complete ACROSTUDY analysis of the full cohort, the use of PEGV monotherapy remained constant over time (55.1% at baseline to 53.3% at year 15), while the use of PEGV in combination with SRL and/or DA decreased (44.9 to 31.0%). This differs slightly from a previous interim analysis of ACROSTUDY, which reported a higher proportion of patients receiving combination therapy over time (20% in 2003 vs 54% in 2012) (23). Differences in these analyses include the number of samples used and how rates were measured, but these final results may reflect the normal clinical course of action in acromegaly treatment. Reasons for the decrease of PEGV in combination with SRL/DA over time are likely multifactorial and varied due to treatment practices across countries and centres, but could be due to toleration issues, patient preference, cost of the combination therapy, radiation, and optimization of biochemical control. Use of combination treatment in acromegaly patients may benefit patients with aggressive acromegaly (6), particularly those partially resistant to first-generation SRLs and with large/invasive tumours (41), similar to those treated with PEGV and pasireotide (42, 43). A recent study showed that low-dose SRL plus weekly PEGV represents a potential novel dosing option for achieving cost-effective, optimal biochemical control in patients with uncontrolled acromegaly requiring combination therapy (44).

PEGV effectiveness may vary depending on patient characteristics. An observational retrospective study found that PEGV resistance was associated with higher BMI and was more frequent with BMI > 30 kg/m^2^ (45). The lower baseline of GH, IGF1 and IGF1 × ULN were associated with disease control, which was more frequent with baseline IGF1 <2.7× ULN (45). Therefore, higher starting PEGV doses and a more rapid up-titration may be necessary for obese patients and in those with IGF1 levels >2.7× ULN (45). The recent Pituitary Society Guidelines suggest that patients with diabetes mellitus and those with a higher BMI require higher doses of PEGV and more rapid up-titration to achieve IGF1 normalization (46).

An earlier, 4-year, longitudinal interim analysis of ACROSTUDY (*n* = 1762) explored the effects of PEGV on glucose metabolism in patients with (*n* = 109) or without diabetes (47). In patients with diabetes mean blood glucose decreased by 20.2 mg/dL from baseline to year 4, while mean HbA1c remained unchanged (47). At year 1, the IGF1 normalization rate was slightly lower in patients with diabetes than those without (52.1% vs 57.4%) (47). Overall, the mean daily PEGV dose was higher in patients with diabetes than without (18.2 mg/day vs 15.3 mg/day) (47). These results were similar to those reported in an 18-study meta-analysis of interventional studies (48), which showed that PEGV alone or combined with SRLs improved glucose metabolism.

Due to the nature of long-term observational, non-interventional studies, ACROSTUDY may have been limited by some underreporting of AEs, such as transient liver enzyme elevation, as data were collected based on routine clinical care and individual schedules. Patients may have started PEGV before ACROSTUDY enrolment, resulting in incomplete baseline information for some patients. Interpretation of pituitary tumour imaging results could also be limited by the protocol design as not all images may have been sent for central assessment.

Patients with acromegaly have significant morbidity and increased mortality if not appropriately treated (2). This study evaluated associated-acromegaly complications in a large cohort in a real-world clinical setting and more than 90% of patients had more than one comorbidity at study entry, with hypertension, diabetes and osteoarthritis being the most frequent ones. Not surprisingly, many patients had colon and thyroid tumours, which highlights the need for screening for these conditions. This large final cohort from ACROSTUDY followed a high number of unselected broad-range of patients from multiple countries for almost 14 years of follow-up, allowing for a better understanding of dose use, efficacy and the safety profile of PEGV and treatment pattern changes over time.

## Conclusions

The global, non-interventional ACROSTUDY provides safety and efficacy data of PEGV on the largest cohort of acromegaly patients with the longest follow-up to date. This review of the final cohort of patients who participated in the study confirms that PEGV improves disease control over time. Safety outcomes with rare tumour progression and infrequent events of liver enzyme elevation are reassuring. Overall, PEGV in this study demonstrated a favourable benefit-to-risk profile for acromegaly treatment both as single and combination therapy. These findings are similar to studies carried out in populations from Japan, Argentina and Brazil.

## Supplementary Material

Supplementary materials

## Declaration of interest

M F is a principal investigator with research support at Oregon Health & Science University for clinical research studies with Crinetics, Novartis, Recordati, Chiasma, Ionis and occasional scientific consultant for Crinetics, Novartis, Pfizer, Ipsen, Ionis, Recordati, and Chiasma. D F is a principal investigator with research support at University Hospital Essen, University of Duisburg-Essen for clinical research studies with Pfizer, Novartis and Ipsen. L d M is a principal investigator for clinical trials for Novartis, Ipsen, Pfizer, and Chiasma. A J v d L received honoraria from Crinetics Inc, Ipsen Pharma, Pfizer Inc, Amolyt Pharma and Tiburion. T B has received honoraria as consultant/speaker, or is a principal investigator for research grants from: Pfizer SAS, Novartis Pharma SAS, Ipsen Pharma, Recordati, Merck-Serono, Sandoz, Novo-Nordisk, Advanz Pharma, and Corcept. J v d L-B, J H-H, C C-H, M P W, S R V, A A P and R G are employees and stockholders of Pfizer. R S is a principal investigator with research support to Johns Hopkins University for clinical research studies with Crinetics, Novartis, and Chiasma and occasional scientific consultant for Ipsen. Maria Fleseriu is on the editorial board of the *European Journal of Endocrinology*. Maria Fleseriu was not involved in the review or editorial process for this paper, on which she is listed as an author.

## Funding

This study was sponsored by Pfizer http://dx.doi.org/10.13039/100004319. Editorial/medical writing support was provided by Hui Zhang, PhD and Dominique Verlaan, PhD, CMPP at Precise Publications, LLC and was funded by Pfizer http://dx.doi.org/10.13039/100004319.
