# Supplementary material for: More than a decade of real-world experience of pegvisomant for acromegaly: ACROSTUDY
Source: Eur J Endocrinol. 2021 Aug 3;185(4):525–38. doi: 10.1530/EJE-21-0239 (PMC8428076; doi:10.1530/EJE-21-0239)
Supplement: Supplementary materials [file supplementary_material.pdf]

Supplementary Appendix

List of Independent Ethics Committees and Institutional Review Boards

Ethik Kommission der Medizinischen Universitat Wien, Wien, Austria; Comité voor Medische Ethiek, Edegem, Belgium; Ethisch Comité, Universitair Ziekenhuis, Gent, Belgium; Conseil National de I’Ordre des Médecins, France; Charité-Universitätsmedizin Berlin, Berlin, Germany; Ethics Committee of the Metaxa-Oncology Hospital, Athens, Greece; Ethics Committee of the General Hospital of Athens, Athens, Greece; Ethics of the Polykliniki Hospital of Athens, Athens, Greece; Egészségugyi Tudományos Tanács Tudományos es Kutatasetikai Bizottsag, Budapest, Hungary; Comitato Etico Delle Aziende Sanitarie dell’Umbria di Perugia, Perugia, Italy; Comitato Etico Dell’Irccs Istituti, Roma, Italy; Comitato Etico Dell’Irccs Ospedale Maggiore Policlinico, Milano, Italy; Comitato Etico Della Provincia di Modena, Modena, Italy; Comitato Etico Dell’Irccs Istituto Auxologico Italiano di Milano, Milano, Italy; Comitato Etico per le Attivita Biomediche Dell Universita Degli Studi Federico II di Napoli; Napoli, Italy; Comitato Etico Dell Universita Cattolica del Sacro Cuore-Policlinico Universitario Agostino Gemelli di Roma, Roma, Italy; Comitato Etico per le Sperimentazione Clinica Della Provincia di Treviso, Treviso, Italy; Comitato Etico Della Provincia di Ferrara, Ferrara, Italy; Comitato Etico Dell’Azienda Policlinico Umberto I di Roma, Roma, Italy; Comitato Etico Dell’Azienda Ospedaliera Universitaria S Giovanni Battista di Torino, Torino, Italy; Comitato Etico Dell´Az.Osp.Univ. Policl. P. Giaccone Dell´Univ. Degli Studi Di Palermo, Palermo, Italy; Comitato Etico Dell´Azienda Ospedaliera Policlinico Consorziale di Bari, Bari, Italy; Comitato Etico per la Sperimentazione Clinica Della Provincia di Treviso, Treviso, Italy; Comitato Etico Dell´Irccs Fondazione S. Raffaele Del Monte Tabor di Milano, Milano, Italy; Comitato Etico-Scientifico Dell´Azienda Ospedaliera Ospedale Niguarda Ca´ Granda di Milano, Milano, Italy;

Comitato Etico per la Sperimentazione Dell´Azienda Ospedaliera di Padova, Padova, Italy; Comitato per la Sperimentazione Clinica Dei Medicinali Dell´A.O.U. Pisana di Pisa, Pisa, Italy; Comitato Etico Dell´Azienda Ospedaliera Universitaria S. Martino di Genova, Genova, Italy; Comitato Bioetica Della Az. Osped. Ospedali Riuniti di Bergamo; Bergamo, Italy; Comitato Etico Indip. Az. Osp-Univ. Policl. S. Orsola-Malpighi Bologna, Bologna, Italy; Comitato Etico per la Sperimentazione Dell´Azienda Ospedaliera di Padova, Padova, Italy; Comitato Etico Dell´Azienda Ospedaliera Universitaria S. Luigi Gonzaga di Orbassano, Orbassano, Italy; Comitato Etico Indipendente Dell'Ospedale Generale Regionale Francesco Miulli di Acquaviva Delle Fonti, Acquaviva Delle Fonti, Italy; Comitato Etico Scientifico Dell´Azienda Ospedaliera Universitaria Policlinico Gaetano Martino di Messina, Messina, Italy; Comitato Etico Azienda Ospedaliera di Rilievo Nazionale E Di Alta Specializzazione Garibaldi di Catania, Catania, Italy; T.A.V. Mw Drs We Vd Voet, Amsterdam, Netherlands; T.A.V. Mw Drs HAM Tebbe, Zwolle, Netherlands; T.A.V. METC, Rotterdam, Netherlands; T.A.V. Mw Mr NMA Verkleij, Leiden, Netherlands; T.A.V. Drs H ’t Hart, Groningen, Netherlands; Instituto Português de Oncologia de Coimbra Francisco Gentil E.P.E., Portugal; Centro Hospitalar Lisboa Ocidental- Hospital Santa Cruz, Portugal; Etická Komisia Pri, Národný Endokrinologický a Diabetologický Ústav, Slovak Republic; CEIC Hospital Universitario Puerta de Hiero Majadahonda, Spain; Regionala Etikprövningsnämnden i Göteborg, Göteborg, Sweden; Central Manchester REC, Manchester, United Kingdom; Chesapeake IRB 6940, Columbia, Maryland, USA; IRB Office Of Research Compliance 8383, Los Angeles, California, USA: University Of Chicago IRB 5841, Chicago, Illinois, USA; VA Loma Linda Healthcare System IRB, Loma Linda, California, USA; Columbia Presbyterian Medical Center IRB, New York, New York, USA; The Institutional Review Board, Cleveland Clinic, Cleveland, Ohio, USA; UCLA Office For Protection Of

Research Subjects, Los Angeles, California, USA; University of Texas MD Anderson Cancer Center IRB, Houston, Texas, USA; Western IRB, Puyallup, Washington, USA; Johns Hopkins Office of Human Subjects Research IRB, Baltimore, Maryland, USA; Wake Forest University Baptist Medical Center, IRB, Winston Salem, North Carolina, USA; Partners Human Research Committee, Boston, Massachusetts, USA; OHSU Research Integrity Office, Portland, Oregon, USA.
